# Relationship between metabolic syndrome and functioning in patients with bipolar disorder type 1

**DOI:** 10.1192/j.eurpsy.2021.1652

**Published:** 2021-08-13

**Authors:** Ö. Şahmelikoğlu Onur, Ö. Baş Uluyol

**Affiliations:** 1 Psychiatry, Bakirkoy Research & Training Hospital for Psychiatry, Neurology and Neurosurgery, İstanbul, Turkey; 2 Psychiatry, Sancaktepe Şehit Prof. Dr. İlhan Varank Research & Training Hospital, İstanbul, Turkey

**Keywords:** bipolar disorder, Metabolic syndrome, Functionality

## Abstract

**Introduction:**

The available literature indicates a possible association between metabolic syndrome (MS) which is highly prevalent among patients with bipolar disorder (BD), and functioning.

**Objectives:**

We sought to compare differences in functional areas of patients with Bipolar Disorder Type 1 (BPD-1)with and without MS in euthymic period.

**Methods:**

This study included 69 euthymic BPD-1 patients without MS and 46 age- and sex-matched BPD-1 patients with MS. All participants completed a sociodemographic form; took the Beck Anxiety Inventory (BAI), Beck Depression Inventory (BDI), Young Mania Rating Scale score, and Bipolar Disorder Functioning Questionnaire. MS was diagnosed according to the International Diabetes Federation (IDF) criteria.

**Results:**

All of the functioning areas were significantly lower in the BPD-1 with MS group than in the without MS group (p < 0.05). Moreover age at onset of disease was significantly lower in BPD-1 group with MS than without MS (p < 0.05). Number of suicide attempts was significantly higher in BPD-1 group with MS than without MS (p < 0.05). Catatonic and melancholic depression were significantly more prevalent in the BPD-1 with MS than without MS (p < 0.05).

**Conclusions:**

Our findings suggest that MS might have an effect on functioning in BD patients even in euthymic period.

**Disclosure:**

No significant relationships.

